# Supplementary material for: Interstadial diversity of East Asian summer monsoon linked to changes of the Northern Westerlies
Source: Nat Commun. 2025 Aug 25;16:7765. doi: 10.1038/s41467-025-63057-2 (PMC12379145; doi:10.1038/s41467-025-63057-2)
Supplement: Supplementary file 1 — Supplementary Information [file 41467_2025_63057_MOESM1_ESM.pdf]

Supporting Information for

Interstadial diversity of East Asian summer monsoon linked to changes of the Northern westerlies

This file includes:

- Supplementary Note 1.1 to 1.6
- Supplementary Tables 1 to 3
- Supplementary Figures 1 to 12
- SI References

Supplementary Notes

1.1 Speleothem $\delta^{18}O$ interpretation

Speleothem $\delta^{18}O$ ($\delta^{18}O_c$) inherits the atmospheric precipitation oxygen isotope composition ($\delta^{18}O_p$) via cave drip water, usually exhibiting similar patterns on millennial-to-orbital timescales from Northeast to Southwest China[1-5]. During the pre-monsoon (March-April-May) season, the Asian summer monsoon (ASM) region receives precipitation from proximal sources, and thus precipitation is characterized by higher values of $\delta^{18}O_p$ (Supplementary Fig. 11a–c). During the summer monsoon season from June to September, moisture from remote sources increases (Fig. 1d; Supplementary Fig. 12b), resulting in lower $\delta^{18}O_p$[3,6] (Supplementary Fig. 11a–c). Modern cave monitoring work in the monsoonal region also demonstrates seasonal isotopic variability in cave drip water $\delta^{18}O$ (e.g., ref. 7,8). On interannual timescale, a lower $\delta^{18}O_c$ indicates a stronger monsoon intensity, with a strengthened large-scale monsoonal circulation and a northward displacement of tropical rain belt[3,9], supported by modeling simulations[10]. These observations imply that the $\delta^{18}O_c$ records from the Indian summer monsoon (ISM) and East Asian summer monsoon (EASM) region on multiple timescales can be broadly interpreted in a framework of overall changes in the upstream convection, moisture source dynamics, and overall monsoon intensity/circulation[1,3,4,10-15].

The South American summer monsoon (SASM) season is in austral summer (December to February). During the SASM season, stronger low-level winds transfer

moisture from the tropical Atlantic across the Amazon basin, as far as southern South America[16]. At centennial-to-orbital timescales, lower (higher) Paraíso $\delta^{18}O_c$ is generally considered as a proxy for stronger (weaker) SASM intensity (overall monsoon circulation) and associated rainfall[1,17].

1.2 Greenland ice-core records and the GICC05 chronology

The Greenland ice-core $\delta^{18}O$ records utilized in this study include NGRIP, GISP2 and GRIP records[18-22]. Greenland ice-core [Ca^{2+}] records include NGRIP, GISP2, GRIP and NEEM records[23-26]. We refer to the Greenland ice-core records ($\delta^{18}O$ and [Ca^{2+}]) on the GICC05 chronology[27-30] with ages reported in thousands of years before 1950 CE (ka BP) or in years before 1950 CE (a BP). We take the reported "Maximum Counting Error" as 2σ (95%) age uncertainty and use the latest nomenclature and timing of interstadials/stadials from ref. 19.

1.3 Uncertainty of the U-Th-based Greenland ice-core chronology

Our study established three tie points between Greenland ice-core and speleothem records (Supplementary Fig. 3; Supplementary Table 1). We combined the maximum tie-point uncertainty in the SN23-1 record (110 years; Supplementary Table 1), the maximum tie-point uncertainty in the ice-core records (110 years; Supplementary Table 1), and the uncertainty regarding the causal correlation between Greenland ice-core [Ca^{2+}] and speleothem $\delta^{18}O$ [estimated to 20 years; following ref. 31], resulting in a combined uncertainty of 160 years (2σ). It is worth noting that the tie point uncertainty of the SN23-1 record is already a combined uncertainty that includes the age model uncertainty and the change point uncertainty (Supplementary Table 1; see Methods).

1.4 Correlation of Antarctic ice-core gas chronology to the improved Greenland chronology

During the last glacial period, the atmospheric CH_4 was primarily associated with tropical and subtropical wetland emissions[32,33]. Greenland temperature (as reflected by ice-core $\delta^{15}N$) leads CH_4 concentrations by 4.5^{+21}_{-24} years during the onset of the Bølling transition[34], while other studies suggest that $\delta^{15}N$-based Greenland temperature leads

CH_4 increases by an average of 56 ± 38 years or 25–70 years over several DO events[35,36]. To combine these scenarios, Buizert et al. [37] proposed a 25-year lag between the midpoints in the West Antarctic Ice Sheet Divide ice core (WDC) CH_4 and the North Greenland Ice-core Project (NGRIP) $\delta^{18}O$ transitions, allowing for a direct alignment of WDC CH_4 and NGRIP $\delta^{18}O$. Based on a comparison of the NGRIP-based GICC05 ice-core chronology with the U-Th-dated Hulu cave speleothem record, a scaling of GICC05 was proposed, and therefore, 1.0063×GICC05 was applied to the WDC gas record over the interval 67.8–31.2 ka[37]. This scaling yields a ca. +350-year shift at 55 ka BP for GICC05, which agrees with the timescale proposed in this study (+230-year shift) within uncertainty.

The match points between WDC CH_4 and NGRIP $\delta^{18}O$ on their original chronologies (WD2014 and GICC05, respectively) are shown in Supplementary Table 2. In order to obtain a common chronology and given that GICC05 has been shifted by +230 years in this study, the WD2014 gas chronology should be adjusted accordingly during our studied time interval. Notably, the time lag between the midpoints in the WDC CH_4 and NGRIP $\delta^{18}O$ transitions has recently been revised to be 29 years[38]. Taken these factors into consideration, we calculated the new match point ages in WDC CH_4 and NGRIP $\delta^{18}O$ (Supplementary Table 2; Supplementary Fig. 3e), thus obtaining the adjusted Antarctic ice-core gas timescale in our studied time interval and are provided in Supplementary Data 3.

1.5 Marine sediment records

Cariaco Basin and Arabian Sea sediment reflectance records were on the GICC05 chronology[39]. In view of the improvement of the GICC05 chronology in our study, we shifted these records by +230 years (Supplementary Fig. 4g, h). For the North Atlantic Pa/Th record, the chronology remains unchanged (Fig. 2a).

1.6 Hemispheric imprints of other centennial-scale events

Another pair of centennial-scale events, DO-14d–e, has also been captured by several proxy records (Supplementary Fig. 4d–j) with an interhemispheric "monsoon seesaw pattern" (Supplementary Fig. 4e, f, i) in the context of ITCZ shifts northward

and then southward (Supplementary Fig. 4h). Intriguingly, a discrepancy among proxy records is apparent between different regions in terms of relative values between the cool phase of interstadial 14d and stadial 15.1. After the warm phase of DO-14e, the cool phase of DO-14d in Greenland ice-core $\delta^{18}O$ (Supplementary Fig. 4d) and [Ca^{2+}] (Supplementary Fig. 3b, c), ASM speleothem (Supplementary Fig. 4e, f) and Arabian Sea reflectance (Supplementary Fig. 4g) records reached a level that is notably higher than Stadial 15.1 but lower than the adjacent interstadials. In contrast, the Cariaco reflectance record and the SASM speleothem record, derived from the tropical Atlantic sector (Supplementary Fig. 1) reached a level during the cool phase of 14d equivalent to stadial 15.1 value (Supplementary Fig. 4h, i). This difference may provide a clue for the potential sources of atmospheric CH_4. During the last glacial period, atmospheric CH_4 was primarily associated with tropical and subtropical wetland emissions[32-34], but it remains difficult to ascertain the exact sources of CH_4 emission. Proxy record comparison shows that the CH_4 change is quite different from proxy records in the tropical Atlantic sector (Supplementary Fig. 4h–j), which suggests that the CH_4 emission from tropical Atlantic wetlands might not be the major control or source of atmospheric CH_4, at least for this time period.

Supplementary Tables

Supplementary Table 1 Age and combined uncertainties for speleothem and Greenland tie points

Speleothem record	Search time window	Age model uncertainty (years)	Change point uncertainty (years)	Age and combined uncertainty (a BP)	Passed the sensitivity tests? Yes (Y)/No (N)
SN23-1 $\delta^{18}O$	55,000–55,250	100	20	55,140 ± 100	Y
SN23-1 $\delta^{18}O$	55,600–55,750	90	50	55,660 ± 100	Y
SN23-1 $\delta^{18}O$	55,850–56,200	110	20	55,970 ± 110	Y

Below are age and combined uncertainties for Greenland ice-core [Ca^{2+}] tie points

Tie-points in speleothem and uncertainty (a BP)	Ice cores	Search time window	Tie points in ice-cores and uncertainty (a BP)	Combined timing for ice-core tie-points
55,140 ± 100	NGRIP	54,930–55,350	55,110 ± 30	55,119 ± 90
	GRIP		55,120 ± 40	
	GISP2		55,100 ± 70	
	NEEM		55,130 ± 30	
55,660 ± 100	NGRIP	55,570–55,750	55,620 ± 10	55,623 ± 20
	GISP2	55,580–55,760	55,620 ± 10	
	NEEM	55,590–55,730	55,630 ± 10	
55,970 ± 110	NGRIP	55,900–56,250	56,030 ± 40	56,025 ± 110
	GRIP		56,060 ± 30	
	GISP2		55,980 ± 30	
	NEEM		56,080 ± 90	

In cases where the plus and minus errors are asymmetrical, the maximum errors were taken. The combined uncertainty is the square root of the age model uncertainty and change point uncertainty (Methods).

The combined timing is the weighted average age calculated based on IsoplotR software[40], and their uncertainties are obtained via quadratically combining the tie point uncertainties from the four ice cores.

All errors are 2σ, and we refer to Greenland and speleothem ages in years before 1950 CE (a BP). The errors that are less than 10 years are listed as 10 years. Greenland ice-core records are on the GICC05+230-year chronology.

Supplementary Table 2 Ages for new match point in NGRIP $\delta^{18}O$ and WDC CH_4

NGRIP match point on GICC05	WDC CH_4 match point on WD2014	NGRIP match point age on GICC05+230	Targeted WDC match point age	WD2014 gas age shift
54,164	54,480	54,394	54,365	−115
54,850	55,170	55,080	55,051	−119
54,940	55,261	55,170	55,141	−120
55,369	55,693	55,599	55,570	−123
55,737	56,063	55,967	55,938	−125
56,555	56,887	56,785	56,756	−131

NGRIP and WDC match point age on their original chronologies are from ref. 37.

The targeted WDC match point age is equal to the value in the third column minus 29 (Supplementary Note 1.4).

Supplementary Table 3 Asian summer monsoon domain speleothem sample dating information

Speleothem name.	Growth length (mm)	Growth rate	Isotope resolution (mm)	Dating resolution (mm)	Average dating error (years)
SN23-1 (this study)	178	0.077 mm/a	0.8	10	140
YX-55[41]	25	0.011 mm/a	0.7	15	290
YX-51[41]	71.5	0.03 mm/a	0.7	17	210
Wu-26[42]	150	0.063 mm/a	1.5	180	200
BT-2[43]	69.5	0.029 mm/a	0.5	12	350

The growth length corresponds to 57–54.65 ka.

Supplementary Figures

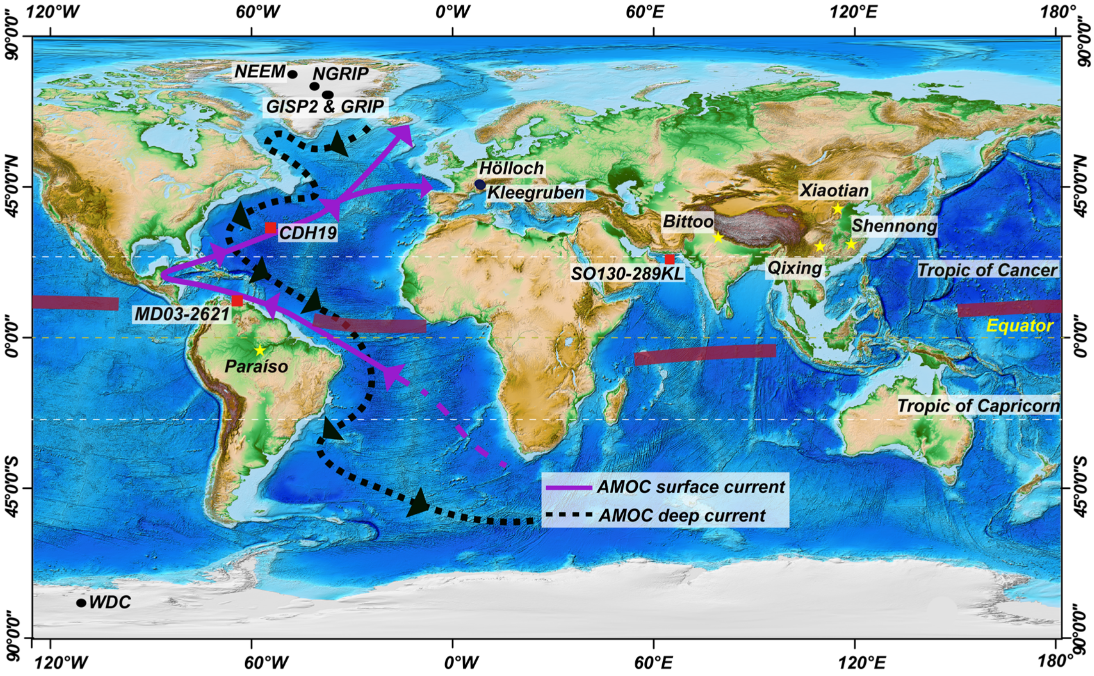

Supplementary Fig. 1. Proxy record locations. Yellow stars show locations of the caves presented in this study. Black dots indicate Greenland and Antarctic ice-core locations and other cave locations discussed in the main text. Red squares delineate marine sediment core locations. Red bars within the tropics indicate the annual-mean position of the Intertropical Convergence Zone (ITCZ) over the oceans[44]. The map was obtained from https://www.ngdc.noaa.gov/mgg/global/. AMOC: Atlantic Meridional Overturning Circulation.

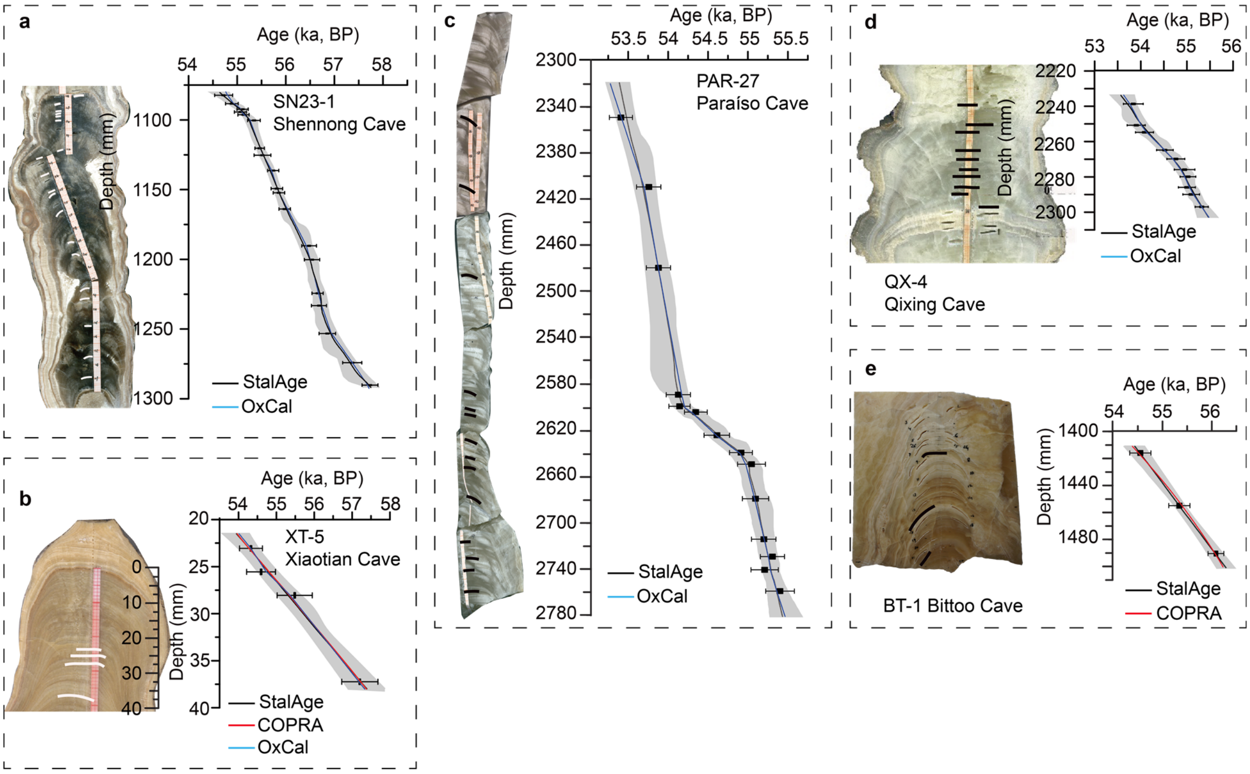

Supplementary Fig. 2. Age models and slabbed images of speleothems. (**a**)–(**e**) Scan images and age models of the speleothem SN23-1 (1,075–1,288 mm), XT-5 (the upper 40 mm), PAR-27 (2,270–2,780 mm), QX-4 (2,220–2,330 mm) and BT-1 (1,410–1,500 mm). The white/black bars indicate positions of subsamples for U-Th dating (Supplementary Data 1). The curves show modeled mean values using different age-modeling algorithms (color-coded)[45-47], the gray bands depict the 95.4% confidence interval using StalAge algorithm[45]. Error bars on U-Th dates represent 2σ analytical error.

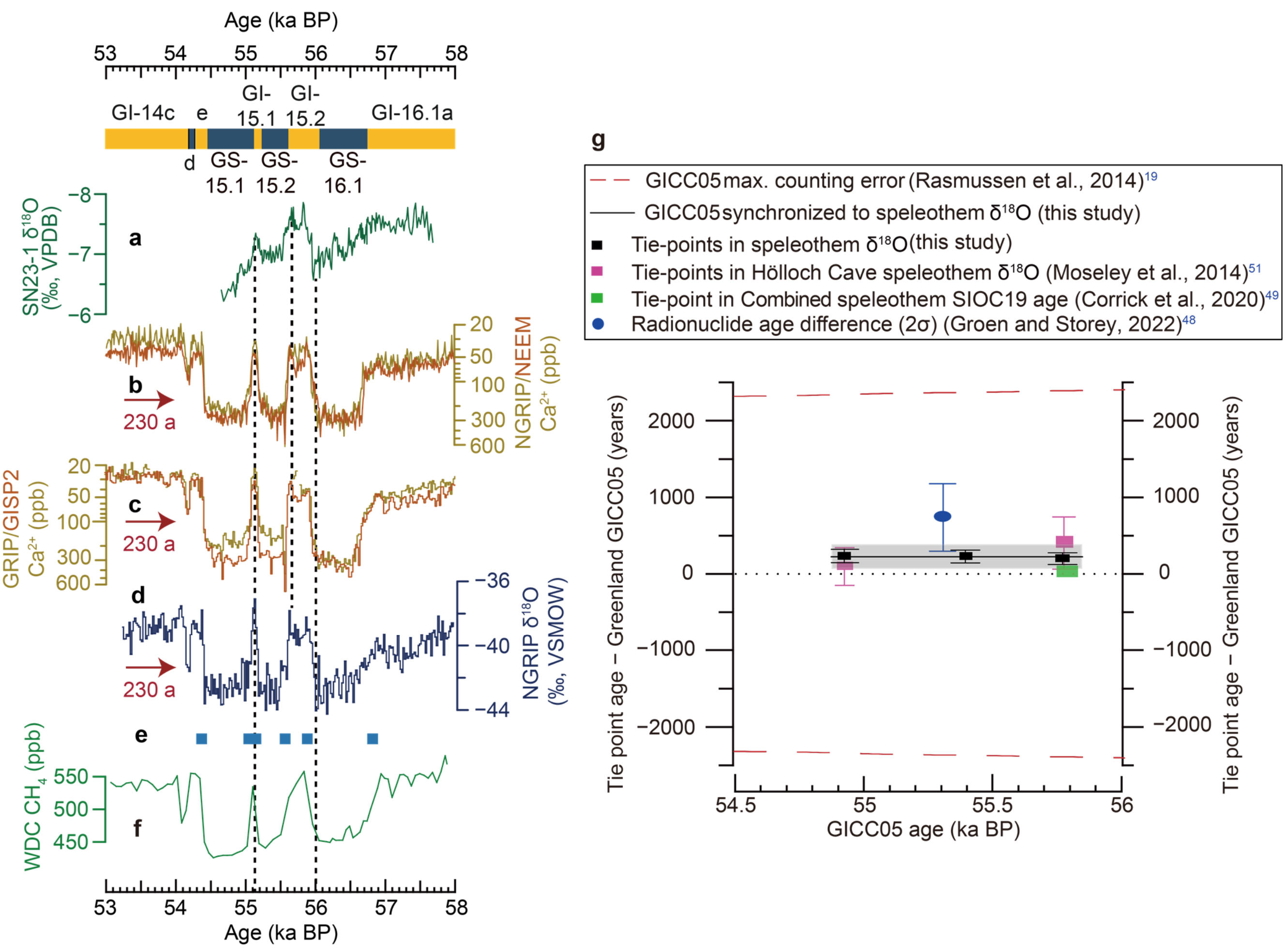

Supplementary Fig. 3. Correlation between bipolar ice-core records and speleothem records. (**a**) speleothem $\delta^{18}O$ record from Shennong cave (this study). (**b**) Greenland NGRIP[23] and NEEM[26] ice-core [Ca^{2+}] record, respectively. (**c**) Greenland GRIP[24] and GISP2[25] ice-core [Ca^{2+}] record, respectively. (**d**) NGRIP ice-core $\delta^{18}O$ record[18,19]. Greenland ice-core records are plotted on the GICC05 chronology (Supplementary Note 1.2) shifted by +230 years via correlating the tie points (vertical dashed lines) between ice-core [Ca^{2+}] records and Shennong cave speleothem $\delta^{18}O$ record (see Methods). (**f**) Antarctic WDC ice-core CH_4 record[32], plotted on the WD2014 chronology and has been tuned to the NGRIP ice-core chronology (GICC05 +230 a) through the tie points shown by the blue squares in (**e**) (Supplementary Table 2; Supplementary Note 1.4). (**g**) Error bars depict the tie points between speleothem and Greenland ice-core records and associated uncertainties (2σ). The gray shading depicts the 95.4% confidence interval of the improved Greenland chronology. The blue dot shows the age difference between the $^{40}Ar/^{39}Ar$ age of the North Atlantic Z2 ash forming Icelandic Thórsmörk peralkaline rhyolitic eruption and the GICC05 age of the Z2 ash layer[48]. The green dot depicts the tie point in Speleothem Interstadial Onset Compilation data set (SIOC19) age[49]. Greenland Stadials (GS) and Greenland Interstadials (GI)[19] are shown at the top left. Cave and core locations are shown in Supplementary Fig. 1.

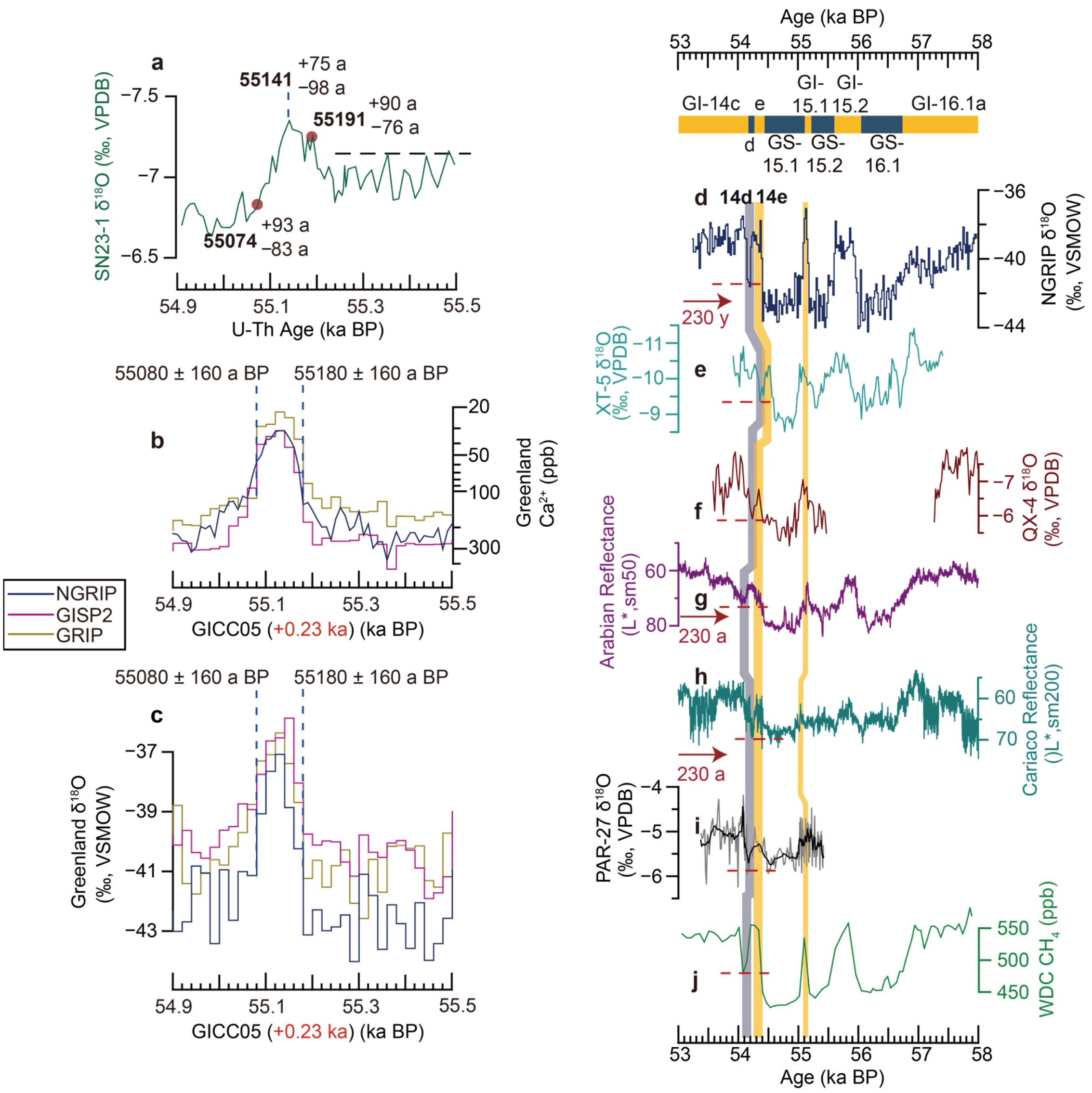

Supplementary Fig. 4. Comparison between speleothem, Greenland ice-core and marine sediment records. (**a**) – (**c**) Comparison between speleothem and ice-core records during DO-15.1. (**a**) Shennong cave speleothem SN23-1 $\delta^{18}O$ record (this study), the red dots indicate the start and end timings of DO-15.1 in the Asian summer monsoon domain (Methods), and the horizontal dashed line illustrates the stadial level prior to DO-15.1. (**b**) Greenland ice-core [Ca^{2+}] records. The vertical dashed lines show the start and end timings of Greenland Interstadial 15.1, determined by Rasmussen et al. [19], the uncertainties were derived from this study (Supplementary Note 1.3). Note the inverted logarithmic y-axis. (**c**) is as same as in (**b**) but for Greenland ice-core $\delta^{18}O$ records. (**d**) Greenland NGRIP ice-core $\delta^{18}O$ records[18,19] on the GICC05 chronology and shifted by +230 years. (**e**) and (**f**) Speleothem $\delta^{18}O$ record from Xiaotian cave (this study) and Qixing cave (this study) in China, respectively. (**g**) and (**h**) show the reflectance record from Arabian Sea (SO130-289 KL core) and Cariaco Basin (MD03-2621 core), respectively[39]. (**i**) Brazilian speleothem $\delta^{18}O$ record from Paraíso cave (this study). (**j**) Antarctic WDC ice-core CH_4 record[32] on the adjusted chronology (Supplementary Fig. 3f; Supplementary Note 1.4; Supplementary Data 3). Greenland Stadials (GS) and Greenland Interstadials (GI)[19] are shown at the top. The vertical yellow bars indicate the initial warm phase 14e of interstadial 14 and the DO-15.1, respectively. The vertical gray bar indicates the cool phase 14d within interstadial 14. The horizontal dashed red lines

indicate the signal level within the cool phase 14d. Cave and core locations are shown in Supplementary Fig. 1.

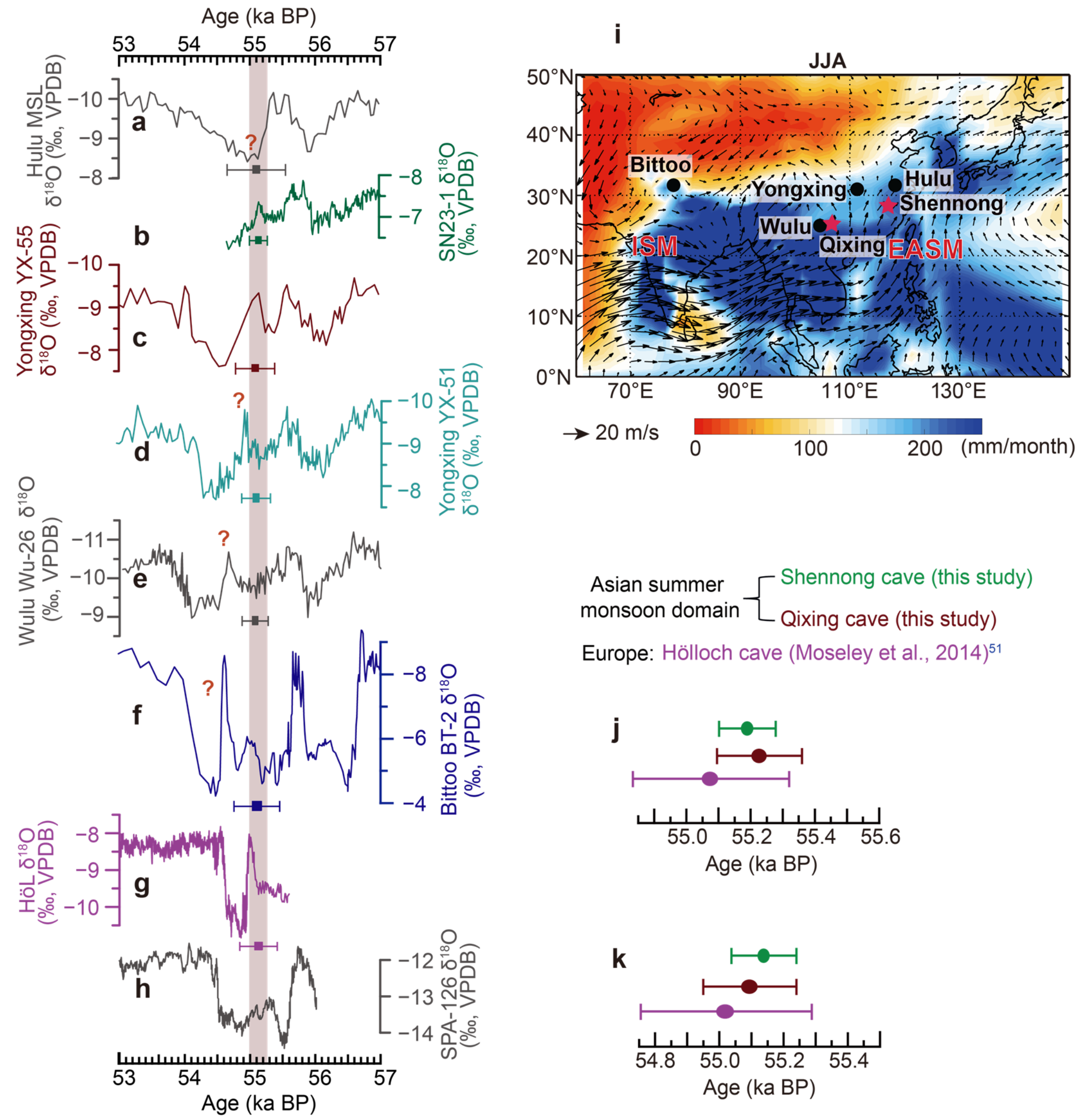

Supplementary Fig. 5. Comparison between Asian summer monsoon domain and European speleothem records. (**a**)–(**f**) Asian summer monsoon domain speleothem $\delta^{18}O$ records from Hulu cave[50], Shennong cave (this study), Yongxing cave[41], Wulu cave[42] in China and Bittoo cave in India[43], respectively. (**g**) and (**h**) European speleothem records from Hölloch cave[51] and Kleegruben cave[52], respectively. The vertical bar indicates the timing of DO15.1 as constrained by our SN23-1 record. Error bars in (**a**)–(**g**) show typical age model uncertainties (2σ) for each record (color-coded). (**i**) Asian summer monsoon domain cave locations. Background is the June-July-August (JJA) monthly spatial rainfall amount (color scale, 1980–2010 CE) and 850 hPa low-level wind (black arrows), data obtained from the Global Precipitation Climatology Project (GPCP) (https://www.esrl.noaa.gov/psd/data/gridded/data.gpcp.html). Red stars show locations for the caves presented in this study, while the black dots indicate locations of other caves mentioned in this study. For European cave locations refer to Supplementary Fig. 1. (**j**) and (**k**) Comparison of the DO-15.1 timing between different regions. (**j**) Comparison of the DO-15.1 onset, where error bars correspond to different cave records (color-coded). SN23-1: 55,191 ± 90 a BP, QX-4: 55,225 ± 135 a BP, Höl: 55,068 ± 250 a

BP. (**k**) same as (**j**) but at the peak of DO-15.1. SN23-1: 55,141 ± 100 a BP, QX-4: 55,095 ± 150 a BP, Höl: 55,018 ± 275 a BP. Note that error bars in (**j**) and (**k**) are combined uncertainties that include the age-model uncertainty as well as change point uncertainty.

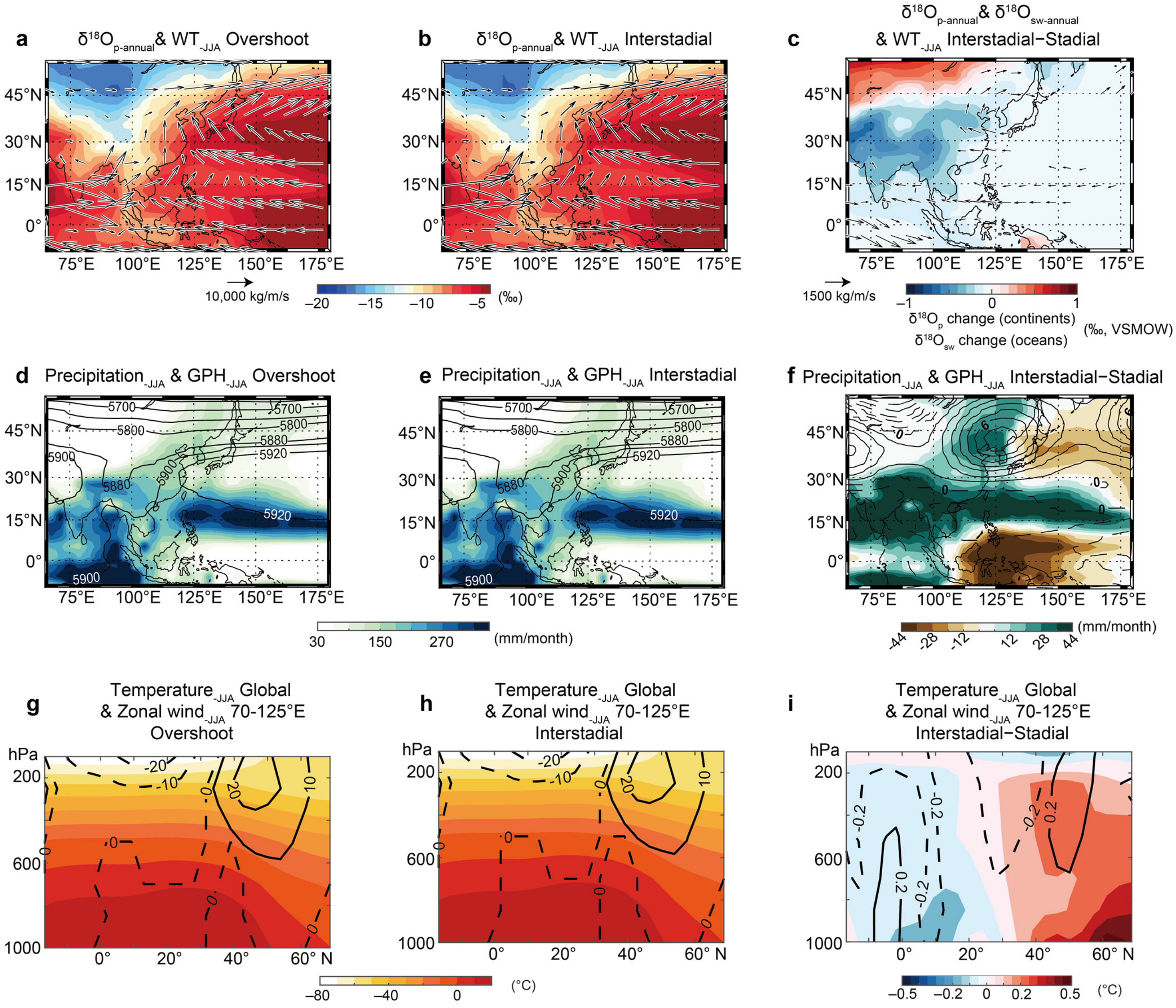

Supplementary Fig. 6. Simulated hydroclimate changes during DO warmings. (**a**) Composites of changes in mean annual $\delta^{18}O_p$ over continents and mean annual $\delta^{18}O$ of sea-surface water ($\delta^{18}O_{sw}$) over oceans (shaded, units: ‰) and water-vapor transport (WT) (vectors, units: kg/m/s) during short (simulated Overshoot phases) DO warmings. (**b**) Same as (**a**), but for long (simulated Interstadial phases) DO warmings. (**c**) Same as (**a**), but for differences between long interstadials and stadials (Interstadial minus Stadial phases). (**d**)–(**f**) are as same as (**a**)–(**c**), respectively, but for summer precipitation (shading, units: mm/month) and 500 hPa geopotential height (lines). (**g**)–(**i**) are as same as (**a**)–(**c**), respectively, but for globally zonal-averaged summer temperature (shadings, °C) and zonally averaged summer wind between 70° and 125° E (lines). The time periods of Stadial, Overshoot and Interstadial phases refer to Fig. 4a. Note that solid lines indicate positive values while dashed lines indicate negative values. DO: Dansgaard-Oeschger. GPH: Geopotential Height.

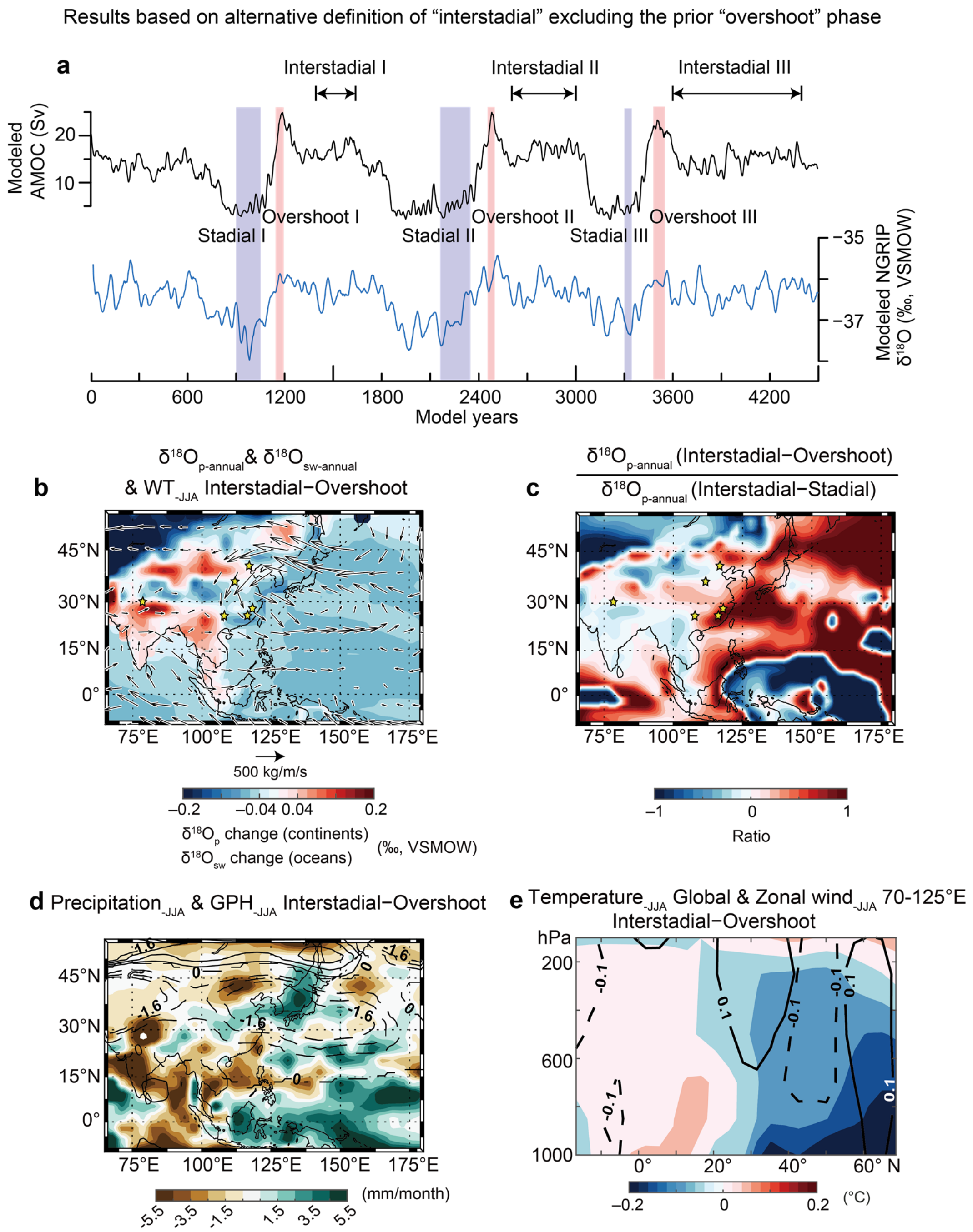

Supplementary Fig. 7. The results based on alternative definition of "interstadial" excluding the prior "overshoot" phase. (**a**) Modeled AMOC index and the NGRIP mean annual $\delta^{18}O_p$ (as same as in Fig. 4a). We classify three AMOC phases: Stadial phases, Overshoot phases and Interstadial phases. The Stadial and Overshoot phases are as same as in Fig. 4a, while the Interstadial phases used here do not include the "Overshoot" phases (Methods). (**b**) Composites of differences in mean annual $\delta^{18}O_p$ over continents and mean annual $\delta^{18}O$ of sea-surface water ($\delta^{18}O_{sw}$) over oceans (shaded, units: ‰) and vertical integrated water-vapor transport (WT) (vectors, units: kg/m/s) between long and short interstadials (Interstadial minus Overshoot phases). (**c**) The proportion of $\delta^{18}O_p$ changes between long and short interstadials (Interstadial minus Overshoot phases) in the long (Interstadial minus Stadial phases) DO warming events. Bittoo, Qixing, Xiaotian, Shennong and Xinglong cave locations are indicated by the yellow stars (refer to Fig. 1d). (**d**) Simulated differences in precipitation (shading, units:

mm/month) and 500 hPa geopotential height (lines) between long and short interstadials (Interstadial minus Overshoot phases). The intervals between each line correspond to $\frac{8}{15}$. (**e**) is as same as in (**d**), but for globally zonal-averaged summer temperature (shadings) and zonal-averaged wind between 70° and 125° E (lines). Note that solid lines indicate positive values while dashed lines indicate negative values. DO: Dansgaard-Oeschger. AMOC: Atlantic Meridional Overturning Circulation.

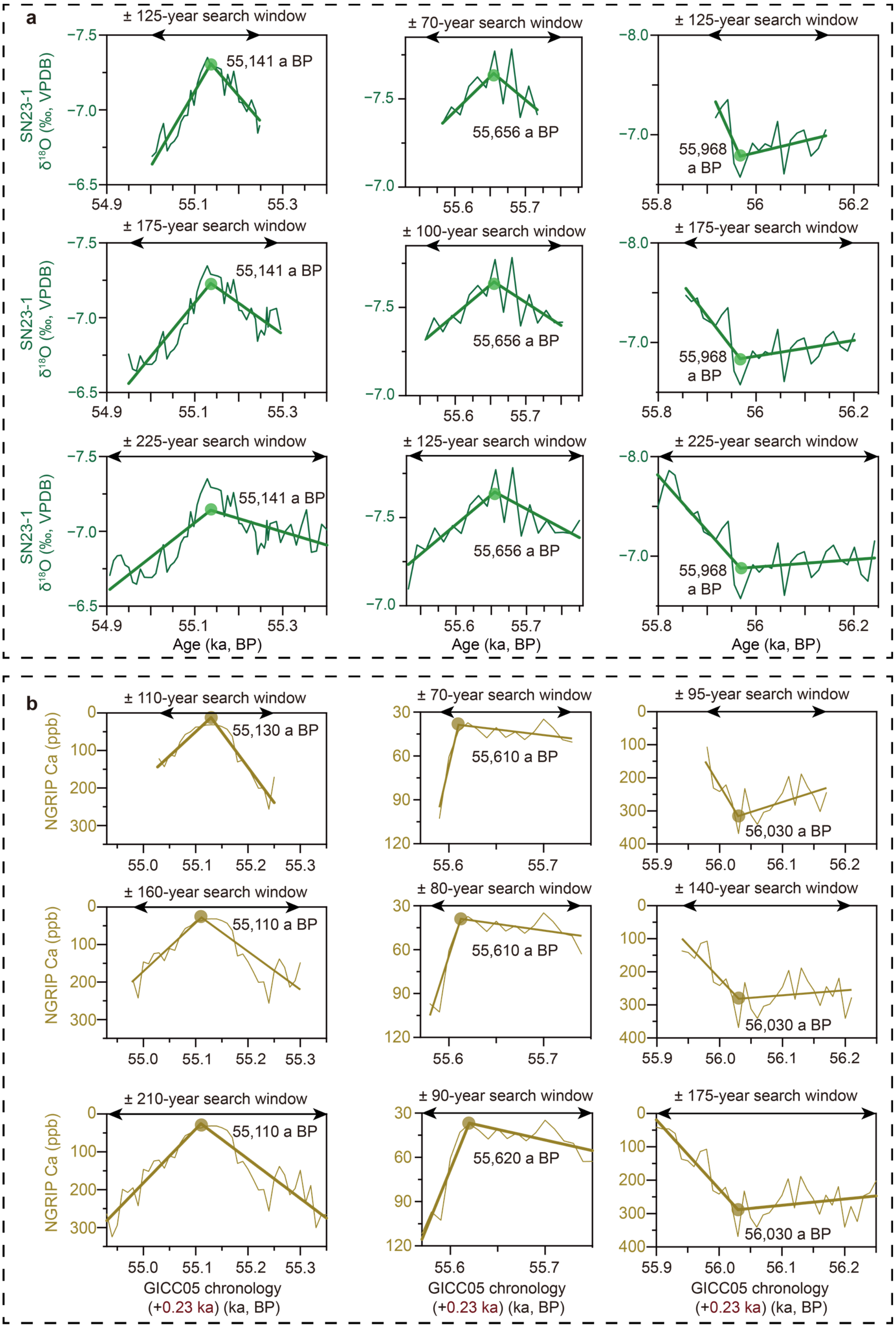

Supplementary Fig. 8. Sensitivity tests of speleothem and ice-core records. The bold ramps are defined by the BREAKFIT algorithm[53] over three search windows of different widths (Methods). The change points are shown and the selected search windows are indicated by the horizontal black arrows.

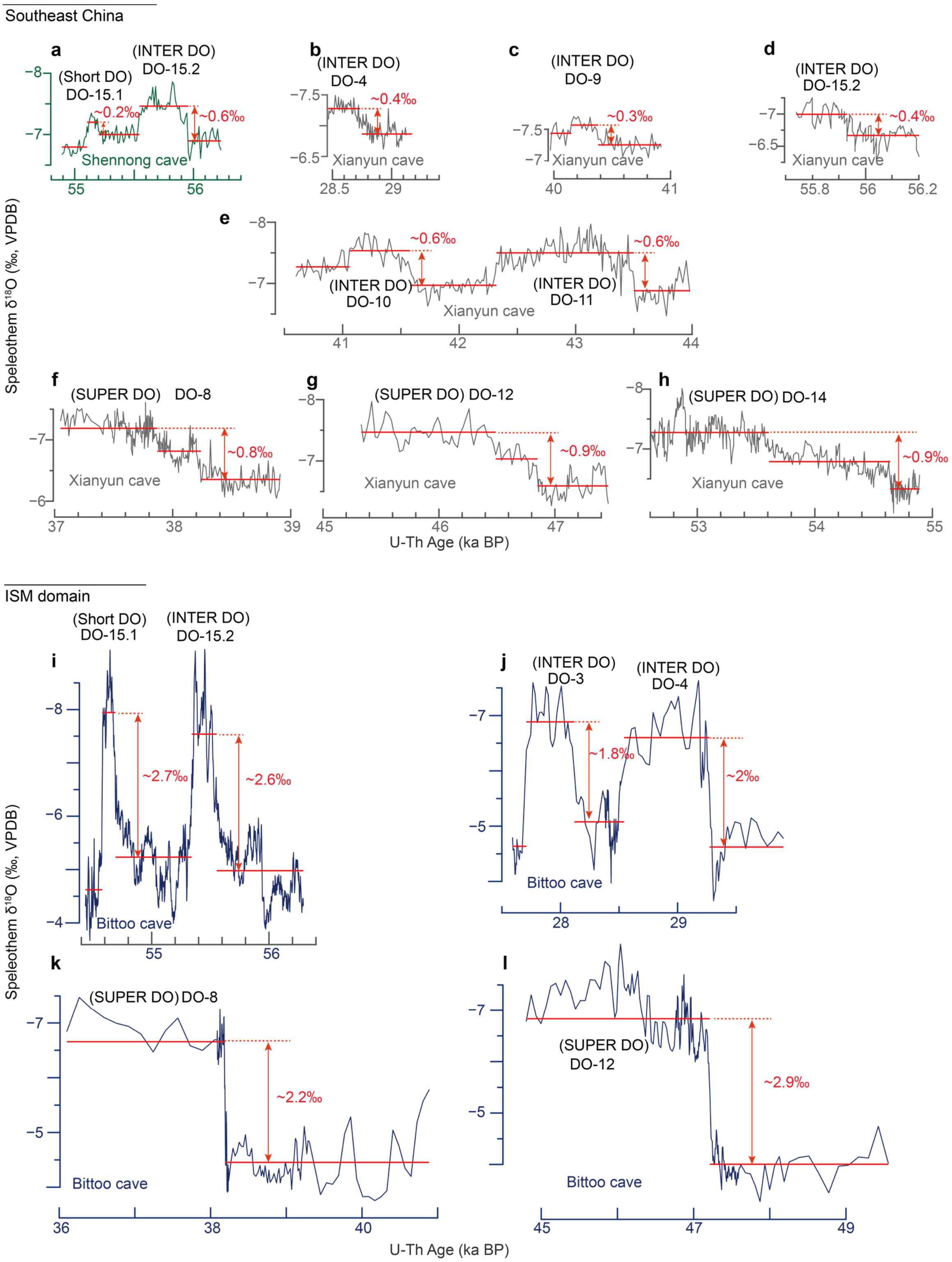

Supplementary Fig. 9. The event amplitude calculated by "Mean-fitting" algorithm. Speleothem $\delta^{18}O$ records from (**a**) Shennong cave (this study) and (**b**)–(**h**) Xianyun cave[54-58] from Southeast China, respectively. (**i**) –(**l**) Indian summer monsoon (ISM) domain speleothem records from Bittoo cave (this study and ref. [43]). The "Mean fitting" algorithm is described in Methods. The vertical red lines show the pre-transition and post-transition levels given by the algorithm; the double-sided arrows illustrate the amplitude. DO: Dansgaard-Oeschger. SUPER DO: Super-long DO; INTER DO: Intermediate-long DO (refer to Fig. 3).

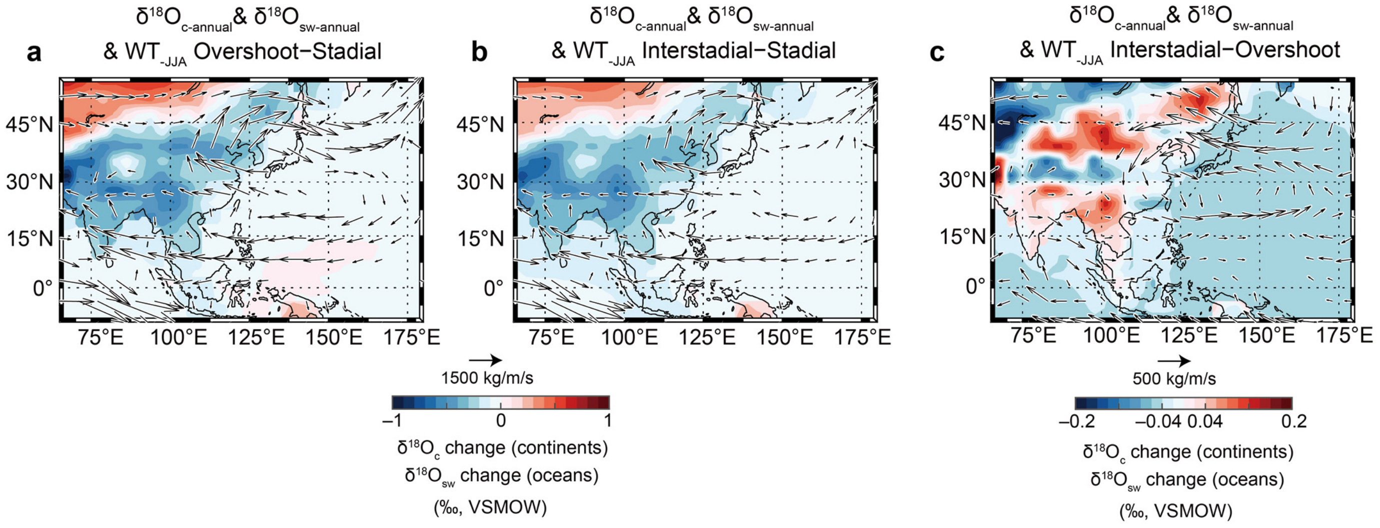

Supplementary Fig. 10. Simulated speleothem and sea-surface water δ^{18}O and water-vapor transport during long and short DO warming events. (**a**)–(**c**) Composites of changes in annual mean cave speleothem δ^{18}O (δ^{18}O$_c$) over continents and annual mean δ^{18}O of sea-surface water (δ^{18}O$_{sw}$) over oceans (shaded, units: ‰) (Methods) and water-vapor transport (WT) (vectors, units: kg/m/s) during simulated short (Overshoot minus Stadial phases) and long (Interstadial minus Stadial phases) DO warming events, and the difference between long and short interstadials (Interstadial minus Overshoot phases). Note that the color scales in (**a**) and (**b**) are different from (**c**). The time periods of Overshoot and Interstadial phases refer to Fig. 4a.

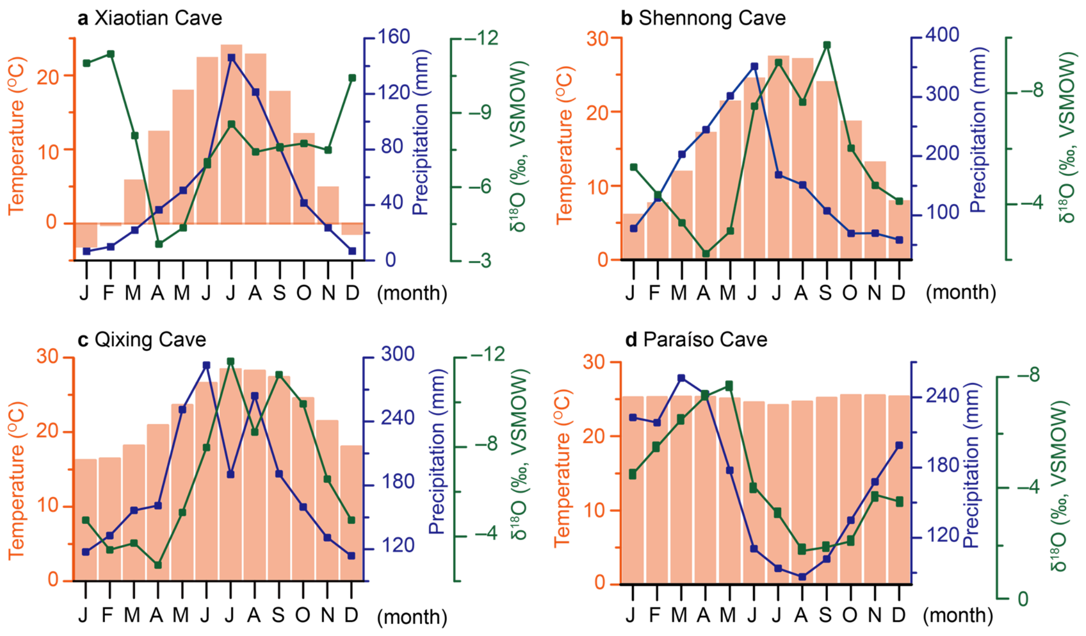

Supplementary Fig. 11. Climatology of the cave sites. Yellow columns and blue lines in (**a**)–(**d**) illustrate the average temperature and precipitation over different cave sites, respectively. Green line and dots in (**a**)–(**d**) indicate precipitation $\delta^{18}O$ ($\delta^{18}O_p$) from Shijiazhuang meteorological station during 1985–2003 CE, $\delta^{18}O_p$ from Changsha meteorological station during 1988–1992 CE, $\delta^{18}O_p$ from Guiyang meteorological station during 1988–1992 CE and $\delta^{18}O_p$ from Manaus during 1998–2010 CE[17], respectively. Temperature and precipitation data for 1951–2020 CE are from Climate Research Unit Time Series version 4.06 (CRU-TS4.06) gridded observation dataset, which has a horizontal resolution of 0.5° × 0.5°[59]. $\delta^{18}O_p$ data are from the International Atomic Energy Agency (IAEA) Global Network of Isotopes in Precipitation (GNIP) database (http://www.iaea.org).

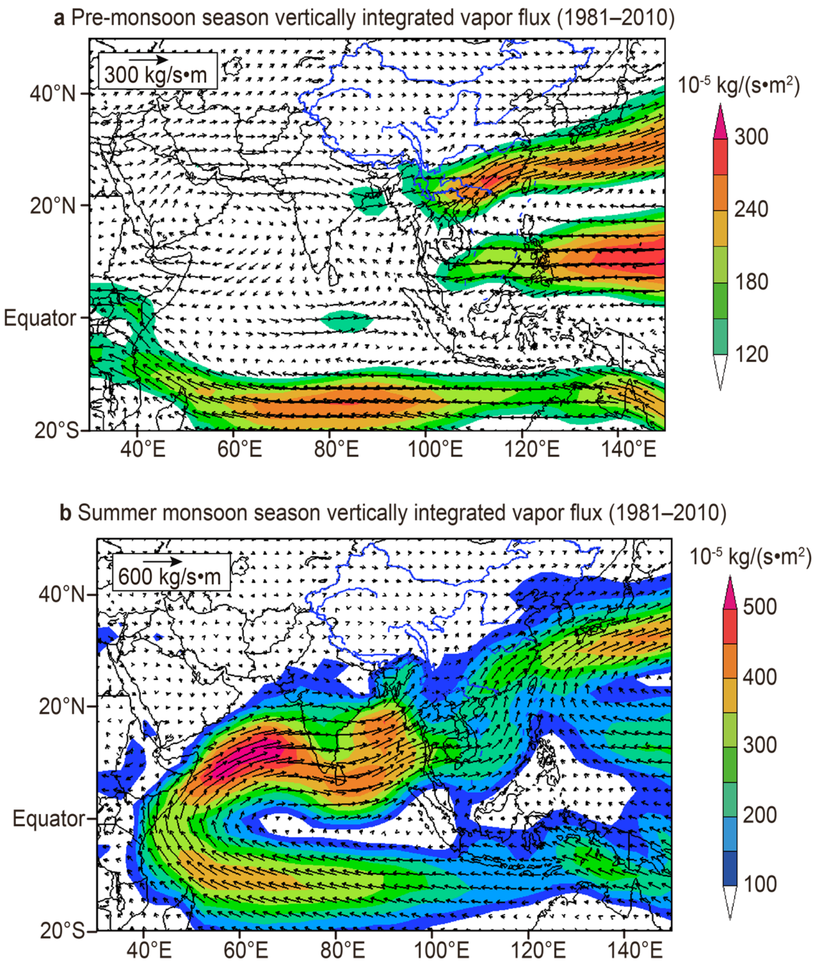

Supplementary Fig. 12. Vertically integrated vapor flux in the Asian summer monsoon domain. (**a**) Pre-monsoon season (March 15 to May 15), and (**b**) monsoon season (June 1 to July 15) during 1981-2010 CE. The background figure was generated using climate data obtained from the Nation Climate Centre of China (http://cmdp.ncc-cma.net/Monitoring/cn_stratosphere_interaction.php?cat=water) and was further modified in this study.

Supplementary references

1. Cheng, H. et al. Onset and termination of Heinrich Stadial 4 and the underlying climate dynamics. *Commun Earth Environ* **2**, 230 (2021).
2. Cheng, H. et al. Orbital-scale Asian summer monsoon variations: Paradox and exploration. *Sci. China-Earth Sci.* **64**, 529-544 (2021).
3. Cheng, H. et al. Chinese stalagmite paleoclimate researches: A review and perspective. *Sci. China-Earth Sci.* **62**, 1489-1513 (2019).
4. Zhang, H. et al. A data-model comparison pinpoints Holocene spatiotemporal pattern of East Asian summer monsoon. *Quat. Sci. Rev.* **261**, 106911, (2021).
5. Zhao, J. et al. New insights towards an integrated understanding of NE Asian monsoon during mid to late Holocene. *Quat. Sci. Rev.* **254**, 106793 (2021).
6. Cheng, H. et al. A penultimate glacial monsoon record from Hulu Cave and two-phase glacial terminations. *Geology* **34**, 217-220 (2006).
7. Duan, W. et al. The transfer of seasonal isotopic variability between precipitation and drip water at eight caves in the monsoon regions of China. *Geochim. Cosmochim. Acta* **183**, 250-266 (2016).
8. Zhao, J. et al. The seasonally altered atmosphere moisture circulations with rainfall and rainfall isotopes in Southwest China. *Front. Earth Sci.* **10** (2022).
9. Chiang, J. C. H., Herman, M. J., Yoshimura, K. & Fung, I. Y. Enriched East Asian oxygen isotope of precipitation indicates reduced summer seasonality in regional climate and westerlies. *Proc. Natl Acad. Sci. USA* **117**, 14745-14750 (2020).
10. Kathayat, G. et al. Interannual oxygen isotope variability in Indian summer monsoon precipitation reflects changes in moisture sources. *Commun Earth Environ* **2**, 96 (2021).
11. Cai, Z., Tian, L. & Bowen, G. J. Spatial-seasonal patterns reveal large-scale atmospheric controls on Asian Monsoon precipitation water isotope ratios. *Earth Planet. Sci. Lett.* **503**, 158-169 (2018).
12. He, C. et al. Hydroclimate footprint of pan-Asian monsoon water isotope during the last deglaciation. *Sci. Adv.* **7**, eabe2611 (2021).
13. Hu, J., Emile-Geay, J., Tabor, C., Nusbaumer, J. & Partin, J. Deciphering oxygen isotope records From Chinese speleothems with an isotope-enabled climate model. *Paleoceanogr. Paleoclimatol.* **34**, 2098-2112 (2019).
14. Liu, X. et al. New insights on Chinese cave $\delta^{18}O$ records and their paleoclimatic significance. *Earth-Sci. Rev.* **207**, 103216 (2020).
15. Tabor, C. R. et al. Interpreting precession-driven $\delta^{18}O$ variability in the South Asian monsoon region. *J. Geophys. Res.-Atmos.* **123**, 5927-5946 (2018).
16. Cheng, H. et al. Climate change patterns in Amazonia and biodiversity. *Nat Commun* **4**, 1411 (2013).
17. Wang, X. et al. Hydroclimate changes across the Amazon lowlands over the past 45,000 years. *Nature* **541**, 204-207 (2017).
18. North Greenland Ice Core Project Members. High-resolution record of Northern Hemisphere climate extending into the last interglacial period. *Nature* **431**, 147-151 (2004).
19. Rasmussen, S. O. et al. A stratigraphic framework for abrupt climatic changes during the Last Glacial period based on three synchronized Greenland ice-core records: refining and extending the INTIMATE event stratigraphy. *Quat. Sci. Rev.* **106**, 14-28 (2014).
20. Grootes, P. M. & Stuiver, M. Oxygen 18/16 variability in Greenland snow and ice with 10^{-3}- to 10^{5}-year time resolution. *J. Geophys. Res.-Oceans* **102**, 26455-26470 (1997).
21. Johnsen, S. J. et al. The $\delta^{18}O$ record along the Greenland Ice Core Project deep ice core and the

problem of possible Eemian climatic instability. *J. Geophys. Res.-Oceans* **102** (1997).
22. Stuiver, M. & Grootes, P. M. GISP2 oxygen isotope ratios. *Quat. Res.* **53**, 277-284 (2000).
23. Erhardt, T. et al. High-resolution aerosol concentration data from the Greenland NorthGRIP and NEEM deep ice cores. *Earth Syst. Sci. Data* **14**, 1215-1231 (2022).
24. Fuhrer, K., Neftel, A., Anklin, M. & Maggi, V. Continuous measurements of hydrogen peroxide, formaldehyde, calcium and ammonium concentrations along the new grip ice core from summit, Central Greenland. *Atmospheric Environment. Part A. General Topics* **27**, 1873-1880 (1993).
25. Mayewski, P. A. et al. Major features and forcing of high-latitude northern hemisphere atmospheric circulation using a 110,000-year-long glaciochemical series. *J. Geophys. Res.-Oceans* **102**, 26345-26366 (1997).
26. Schüpbach, S. et al. Greenland records of aerosol source and atmospheric lifetime changes from the Eemian to the Holocene. *Nat Commun* **9**, 1476 (2018).
27. Andersen, K. K. et al. The Greenland Ice Core Chronology 2005, 15–42ka. Part 1: constructing the time scale. *Quat. Sci. Rev.* **25**, 3246-3257 (2006).
28. Rasmussen, S. O. et al. A new Greenland ice core chronology for the last glacial termination. *J. Geophys. Res.-Atmos.* **111** (2006).
29. Svensson, A. et al. A 60 000 year Greenland stratigraphic ice core chronology. *Clim. Past* **4**, 47-57 (2008).
30. Svensson, A. et al. The Greenland Ice Core Chronology 2005, 15–42ka. Part 2: comparison to other records. *Quat. Sci. Rev.* **25**, 3258-3267, doi:https://doi.org/10.1016/j.quascirev.2006.08.003 (2006).
31. Dong, X. et al. Coupled atmosphere-ice-ocean dynamics during Heinrich Stadial 2. *Nat Commun* **13**, 5867 (2022).
32. Rhodes, R. H. et al. Enhanced tropical methane production in response to iceberg discharge in the North Atlantic. *Science* **348**, 1016-1019 (2015).
33. Riddell-Young, B. et al. Atmospheric methane variability through the Last Glacial Maximum and deglaciation mainly controlled by tropical sources. *Nat. Geosci.* **16**, 1174-1180 (2023).
34. Rosen, J. L. et al. An ice core record of near-synchronous global climate changes at the Bølling transition. *Nat. Geosci.* **7**, 459-463 (2014).
35. Baumgartner, M. et al. NGRIP CH_4 concentration from 120 to 10 kyr before present and its relation to a $\delta^{15}N$ temperature reconstruction from the same ice core. *Clim. Past* **10**, 903-920 (2014).
36. Huber, C. et al. Isotope calibrated Greenland temperature record over Marine Isotope Stage 3 and its relation to CH4. *Earth Planet. Sci. Lett.* **243**, 504-519 (2006).
37. Buizert, C. et al. The WAIS Divide deep ice core WD2014 chronology-Part 1: Methane synchronization (68–31 ka BP) and the gas age–ice age difference. *Clim. Past* **11**, 153-173 (2015).
38. Martin, K. C. et al. Bipolar impact and phasing of Heinrich-type climate variability. *Nature* **617**, 100-104 (2023).
39. Deplazes, G. et al. Links between tropical rainfall and North Atlantic climate during the last glacial period. *Nat. Geosci.* **6**, 213-217 (2013).
40. Vermeesch, P. IsoplotR: A free and open toolbox for geochronology. *Geosci. Front.* **9**, 1479-1493 (2018).
41. Chen, S. et al. Strong coupling of Asian Monsoon and Antarctic climates on sub-orbital timescales. *Sci. Rep.* **6**, 32995 (2016).
42. Liu, D. et al. Sub-millennial variability of Asian monsoon intensity during the early MIS 3 and its analogue to the ice age terminations. *Quat. Sci. Rev.* **29**, 1107-1115 (2010).

43. Kathayat, G. et al. Indian monsoon variability on millennial-orbital timescales. *Sci Rep.* **6**, 24374 (2016).
44. Schneider, T., Bischoff, T. & Haug, G. H. Migrations and dynamics of the intertropical convergence zone. *Nature* **513**, 45-53 (2014).
45. Scholz, D. & Hoffmann, D. L. StalAge – An algorithm designed for construction of speleothem age models. *Quat. Geochronol.* **6**, 369-382 (2011).
46. Breitenbach, S. F. M. et al. COnstructing Proxy Records from Age models (COPRA). *Clim. Past* **8**, 1765-1779 (2012).
47. Ramsey, C. B. & Lee, S. Recent and planned developments of the program OxCal. *Radiocarbon* **55**, 720-730 (2013).
48. Groen, M. & Storey, M. An astronomically calibrated $^{40}Ar/^{39}Ar$ age for the North Atlantic Z2 Ash: Implications for the Greenland ice core timescale. *Quat. Sci. Rev.* **293**, 107526 (2022).
49. Corrick, E. C. et al. Synchronous timing of abrupt climate changes during the last glacial period. *Science* **369**, 963-969 (2020).
50. Cheng, H. et al. The Asian monsoon over the past 640,000 years and ice age terminations. *Nature* **534**, 640-646 (2016).
51. Moseley, G. E. et al. Multi-speleothem record reveals tightly coupled climate between central Europe and Greenland during Marine Isotope Stage 3. *Geology* **42**, 1043-1046 (2014).
52. Spötl, C., Mangini, A. & Richards, D. A. Chronology and paleoenvironment of Marine Isotope Stage 3 from two high-elevation speleothems, Austrian Alps. *Quat. Sci. Rev.* **25**, 1127-1136 (2006).
53. Mudelsee, M. Break function regression. *Eur. Phys. J.-Spec. Top.* **174**, 49-63 (2009).
54. Che, Y.-P., Xiao, H.-Y., Jiang, X.-Y. & Cai, B.-G. Precise dating of the Dansgaard-Oeschger event 4 and its sub-cycles: Evidence from a stalagmite from Xianyun Cave in Longyan City, Fujian Province. *Journal Of Palaeogeography* [in Chinese] **22**, 377-384 (2020).
55. Zhang, X. et al. A gradual transition into Greenland interstadial 14 in southeastern China based on a sub-decadally-resolved stalagmite record. *Quat. Sci. Rev.* **253**, 106769 (2021).
56. Zhang, X., Qiu, W., Jiang, X., Cai, B. & Shen, C.-C. East Asian summer monsoon evolution during Heinrich Stadial 5. *Paleogeogr. Paleoclimatol. Paleoecol.* **654**, 112467 (2024).
57. Zhang, X. et al. Three-phase structure of the East Asia summer monsoon during Heinrich Stadial 4 recorded in Xianyun Cave, southeastern China. *Quat. Sci. Rev.* **274**, 107267 (2021).
58. Li, Y. et al. Millennial-scale East Asian summer monsoon hydroclimate variability during 47–39 kyr B.P. inferred from a stalagmite IRMsoft-flux record in Southeastern China. *Glob. Planet. Change* **253**, 104954 (2025).
59. Harris, I., Osborn, T. J., Jones, P. & Lister, D. Version 4 of the CRU TS monthly high-resolution gridded multivariate climate dataset. *Sci Data.* **7**, 109 (2020).
