# Supplementary material for: Interstadial diversity of East Asian summer monsoon linked to changes of the Northern Westerlies
Source: Nat Commun. 2025 Aug 25;16:7765. doi: 10.1038/s41467-025-63057-2 (PMC12379145; doi:10.1038/s41467-025-63057-2)
Supplement: Supplementary file 2 — Description of Additional Supplementary Files [file 41467_2025_63057_MOESM2_ESM.pdf]

Description of Additional Supplementary Files

File name: Supplementary Data 1
Description: U-Th dating results for 4 speleothems.

File name: Supplementary Data 2
Description: $\delta^{18}O$ time-series for 5 speleothem records.

File name: Supplementary Data 3
Description: Data of the main figures showed in the main text, including the data from referenced papers.

File name: Supplementary Code 1
Description: The "Mean-fitting" algorithm used to determine the event amplitudes.
