# Supplementary material for: Interstadial diversity of East Asian summer monsoon linked to changes of the Northern Westerlies
Source: Nat Commun. 2025 Aug 25;16:7765. doi: 10.1038/s41467-025-63057-2 (PMC12379145; doi:10.1038/s41467-025-63057-2)
Supplement: Supplementary file 3 — Supplementary Code 1 [file 41467_2025_63057_MOESM3_ESM.docx]

% This collection of MATLAB code is a supplement to the paper:

%

% Dong, X. et al., Interstadial diversity of East Asian summer monsoon linked to changes of the

% Northern Westerlies (2025)

% The code performs the “Mean-fitting” analysis

% as described in that work.

%

% To run the code, the user should put the name of .csv file in Line25,

% The file should include two columns (first: age; Second: proxy record) with no title.

% The .csv file should be put in the same folder of this code

% Then the user can set the maximum number of trending lines in Line31

% In case of questions or comments, please contact me on the address below.

% Thanks for your interest in this work.

%

% Kind regards,

%

% Xiyu Dong

%

% Email: xiyu_dong@126.com

% Institute of Global Environmental Change, Xi'an Jiaotong University, Xi’an, 710049, China

%

%

clear all; clc; clf;

series = load ('proxy.csv'); % **the user should put the name of .csv file here**

t=series(:,1);

x=series(:,2);

x=x(1:end);

t=t(1:end);

cp_max=4; %% **the user can set Maximum number of change points here**

figure (1);

line (t,x);

cp= findchangepts(x,'MaxNumChanges',cp_max,'Statistic','mean');

L= length(x);

CL=[1 cp' L];

N=length(CL)-1;

%% calculate the mean value of each pieced time series

for i = 1 : N

m(i) = mean(x(CL(i):CL(i+1)));

end

%% draw the horizontal lines indicating the fitted mean values

for i = 1 : N

line([t(CL(i)) t(CL(i+1))],[m(i) m(i)],'LineWidth',0.75,'Color','red','LineStyle','-');

end

%% draw the vertical lines, i.e., change points

for i=1:length(cp)

line([t(cp(i)) t(cp(i))],[min(x) max(x)],'LineWidth',0.75,'Color','red','LineStyle','--')

end

set (gca,'Ydir','reverse')
